# Cloning and Immunosuppressive Properties of an Acyl-Activating Enzyme from the Venom Apparatus of *Tetrastichus brontispae* (Hymenoptera: Eulophidae)

**DOI:** 10.3390/toxins11110672

**Published:** 2019-11-18

**Authors:** Xiao-Mei Zhang, Hua-Jian Zhang, Min Liu, Bin Liu, Xia-Fang Zhang, Cheng-Jun Ma, Ting-Ting Fu, You-Ming Hou, Bao-Zhen Tang

**Affiliations:** 1 State Key Laboratory of Ecological Pest Control for Fujian and Taiwan Crops, Fujian Agriculture and Forestry University, Fuzhou 350002, Fujian, China; 2 Fujian Provincial Key Laboratory of Insect Ecology, Department of Plant Protection, Fujian Agriculture and Forestry University, Fuzhou 350002, Fujian, China

**Keywords:** 4-coumarate:CoA ligase-like 4, acyl-activating enzyme, parasitoid venom, host, parasitoid wasp, parasitization, immunity, *Tetrastichus brontispae*, *Octodonta nipae*

## Abstract

Venom injected into the host plays vital roles in facilitating successful parasitization and development for parasitoid wasps, especially those devoid of polydnavirus, and the abundant venom proteins appear to be most likely involved in parasitization success. Previously, we found the four most abundant venom proteins, including 4-coumarate:CoA ligase-like 4 (4CL4-like), in the *Tetrastichus brontispae* (Hymenoptera: Eulophidae) venom apparatus. In this study, we cloned, expressed *T. brontispae* 4CL4-like (Tb4CL4-like) in *Escherichia coli*, and investigated its immunosuppressive properties. The deduced amino acid sequence for Tb4CL4-like shares high identity at conserved amino acids associated with the acyl-activating enzyme (AAE) consensus motif but shows only <40% identity with the members in the AAE superfamily. mRNA abundance analysis indicated that Tb4CL4-like was transcribed mainly in the venom apparatus. Recombinant Tb4CL4-like inhibited *Octodonta nipae* (Coleoptera: Chrysomelidae) pupal cellular encapsulation and spreading by targeting the hemocyte cytoskeleton and reduced the hemocyte-mediated phagocytosis of *E. coli* in vivo. Moreover, Tb4CL4-like exhibited greater affinity to palmitic acid and linolenic acid based on the molecular docking assay and is hypothesized to be involved in fatty acid metabolism. In conclusion, our results suggest that Tb4CL4-like may be an immunity-related AAE protein that is involved in the regulation of host immunity through fatty acid metabolism-derived signaling pathways.

## 1. Introduction

Hymenopteran parasitoids are valuable insects in the natural and augmentative biological control of various insect pests. Endoparasitoids deposit their eggs inside the hosts that they use as a source of nutrients for the development of their offspring. Idiobiont endoparasitoids and most koinobiont endoparasitoids usually lead to host death. The deposition of endoparasitoid eggs into insect hosts exposes developing endoparasitoids to host immune responses, mostly melanotic encapsulation [1]. During this encapsulation, hemocytes adhere to the surface of eggs and spread to form multilayers for engulfing eggs, which are ultimately accompanied by melanization [2]. For successful parasitization, endoparasitoids have evolved strategies to evade or counteract these immunological defenses as well as to manipulate host-specific physiology, the most often documented being the injection of venom containing virulence molecules at the time of oviposition [3,4,5]. Venom can act synergistically with other host regulation factors, such as polydnavirus (PDV), in some cases [6]. However, in endoparasitoids devoid of polydnaviruses, venom alone is sufficient to exert adverse effects on the host immune response [1].

Recently, venomic (transcriptomics and/or proteomics) technologies have allowed for significant advances in the knowledge of parasitoid venom composition. Major groups in endoparasitoid venom composition consist of enzymes, protease inhibitors, some immune-related proteins, binding proteins and so on [3,4,7]. Venom proteins are considerably complex and diverse and can evolve quickly and employ new functions to adapt to hosts [4,5,8]. However, venom protein analysis is far from complete. Until now, only a few reports are available regarding the characterizations and functional studies of different venom components. For example, two venom proteins (Vpr1 and Vpr3) from *Pimpla hypochondriaca*, a Rho GTPase-activating protein domain-containing venom protein (P4) from *Leptopilina boulardi*, and calreticulin identified in *Pteromalus puparum* venom were demonstrated to have detrimental effects on host hemocyte function, thus suppressing hemocyte-mediated immune response [9,10,11,12,13]. A serpin protein from *P. puparum* (PpS1V) venom suppresses host prophenoloxidase activation probably by forming a complex with the host hemolymph proteinase PrPAP1 [14]. Extracellular superoxide dismutase from *L. boulardi* venom can also inhibit *Drosophila* host phenoloxidase activity [15]. High amounts of aspartylglucosaminidase secreted in the venom of *Asobara tabida* and *Leptopilina heterotoma* have aspartylglucosaminidase activity and efficient asparaginase, and are likely to play a role in the transient paralysis of host larvae and/or in blocking sensory class IV neurons essential for the host cellular immune response [16].

*Tetrastichus brontispae* Ferriere (Hymenoptera: Eulophidae) is a gregarious pupal endoparasitoid with a narrow host range and mainly prefers to parasitize the one-day-old pupae of two Chrysomelidae beetles of palms, *Brontispa longissima* (Gestro) and *Octodonta nipae* (Maulik). *T. brontispae* Ferriere has been applied as a biological agent to successfully control the population of *B. longissima* in the field in many countries [17]. Its potential to manage *O. nipae* has also been validated in the lab [18]. *T. brontispae* is devoid of polydnavirus, and its venom is the main virulence factor to manipulate the host immunological physiology [19]. We found that its venom extracts had detrimental effects on host encapsulation (unpublished data). Moreover, the four most abundant venom apparatus proteins that are probably abundant venom proteins, including neprilysin-2-like, kynurenine-oxoglutarate transaminase 1-like, 4-coumarate:CoA ligase-like 4 (4CL4-like), and venom protein r-like protein, were identified using label-free quantitative proteomics in combination with a transcriptomic approach [20]. Among these proteins, 4CL4-like, a novel venom protein that has never been reported before or is not deposited in the venom database, belongs to a Class I adenylate-forming enzyme superfamily. This group of enzymes is also termed the acyl-activating enzyme (AAE) or AMP-binding protein superfamily, and is involved in an immense variety of metabolic pathways, such as fatty acid oxidation and enzyme regulation [21,22]. Class I adenylate-forming enzymes comprise aryl- and acyl-CoA synthetases, the adenylation domain of nonribosomal peptide synthetases, methylmalonyl-CoA synthetases, fatty acid-AMP ligases, aryl-CoA ligases, and luciferases [23]. 4-Coumarate:CoA ligases (4CLs) catalyze the activation of 4-coumarate and related substrates to the respective CoA esters and play a vital role in the phenylpropanoid-derived compound pathway [24]. 4CLs have been sequenced from numerous plant species; thus, it is interesting that 4CL4 is recruited and has evolved to perform venom functions. Therefore, in the present study, we mainly investigated whether 4CL4-like from *T. brontispae* (Tb4CL4-like) performs a new venom function and analyzed the immune suppressive properties, especially the effects on host melanotic encapsulation, and the possible mechanisms in immunosuppression.

## 2. Results

### 2.1. Characterization and Sequence, Phylogenetic and Motif Analyses of Tb4CL4-Like

**Figure 1:**
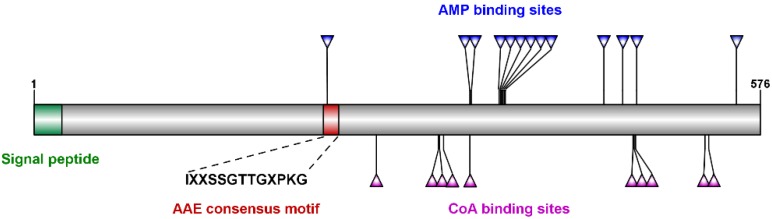
Domain organization of *Tetrastichus brontispae* 4-coumarate:CoA ligase-like 4 (Tb4CL4-like). Tb4CL4-like belongs to the Class I adenylate-forming enzyme superfamily, which contains an acyl-activating enzyme (AAE) consensus motif (IXXSSGTTGXPKG), AMP-binding sites and CoA-binding sites. The predicted signal peptide is also marked on the plot.

Based on the partial nucleotide sequence from *T. brontispae* transcriptome data and the sequencing results of 5′/3′ RACE, the full-length sequence of the Tb4CL4-like gene was obtained. The full-length Tb4CL4-like was 1902 bp, and its open reading frame encoded 576 amino acid residues with a predicted signal peptide consisting of the first 23 residues (Figure 1). There were two and five putative *N*-linked and *O*-linked glycosylation sites (Figure S1), respectively. The predicted molecular mass for the mature Tb4CL4-like protein was 65.28 kDa, with a theoretical isoelectric point of 9.16. The online BLAST tool showed that Tb4CL4-like exhibits the typical characteristics of the Class I adenylate-forming enzyme superfamily, containing an AAE consensus motif (IXXSSGTTGXPKG), AMP-binding sites and CoA-binding sites (Figure 1). This AAE region is very rich in Ser/Thr/Gly that is followed by a conserved Pro–Lys–Gly triplet, and is critical in the adenylation step. However, Tb4CL4-like had only 39.7% identity (the highest identity) to an uncharacterized protein from *Trichomalopsis sarcophagae*, 35.8% identity to the putative 4-coumarate:CoA ligase 3 from *Habropoda laboriosa*, and 35.4% identity to the luciferin 4-monooxygenase from *Apis cerana*.

**Figure 2:**
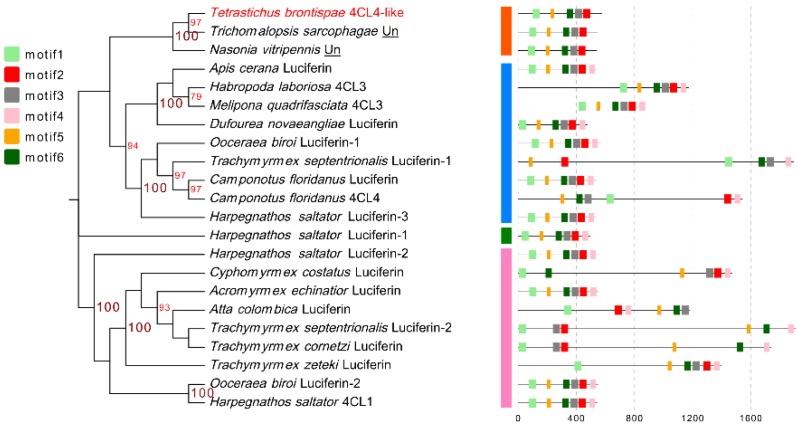
Phylogenetic relationships and architecture of conserved protein motifs in Tb4CL4-like and other Class I adenylate-forming enzyme members. The phylogenetic non-rooted tree was constructed based on the amino acid sequences using MEGA X software with maximum likelihood estimation. The bootstrap values at each branch indicate the percent of 1000 replications, and only those higher than 70 are shown. The underlined “Un” denotes uncharacterized protein. Tb4CL4-like is highlighted in red. The names and accession numbers are summarized in Table S2. The motif composition of Class I adenylate-forming enzyme members is shown on the right panel. The six motifs are displayed in different colored boxes. Clades in motifs corresponding to different orders are shown in different colors. The sequence information for these motifs is provided in Table S1. The length of protein can be judged using the scale at the bottom.

To further test the phylogenetic relationship between Tb4CL4-like and other Class I adenylate-forming enzyme members, 21 proteins with more than 25% identity to Tb4CL4-like from the Universal Protein Resource (UniProt) were retrieved to build a maximum likelihood tree. The dendrogram indicated that Tb4CL4-like and two uncharacterized proteins from *T. sarcophagae* and *Nasonia vitripennis* were clustered together in the same clade, while luciferin 4-monooxygenases and 4CLs were clustered together in three clades (Figure 2 and Figure S2). Moreover, MEME motif analysis representing the structure of these proteins showed that Tb4CL4-like and the two uncharacterized proteins shared highly similar motif arrangements and did not contain motif4 (Figure 2, Table S1). All the other members contained the six motifs (Figure 2 and Table S1). Taken together, the lower identities and the phylogenetic analysis results with the conserved motif arrangements may signify the unique biological role of Tb4CL4-like.

### 2.2. Transcription Profiles of Tb4CL4-Like in Different Tissues

**Figure 3:**
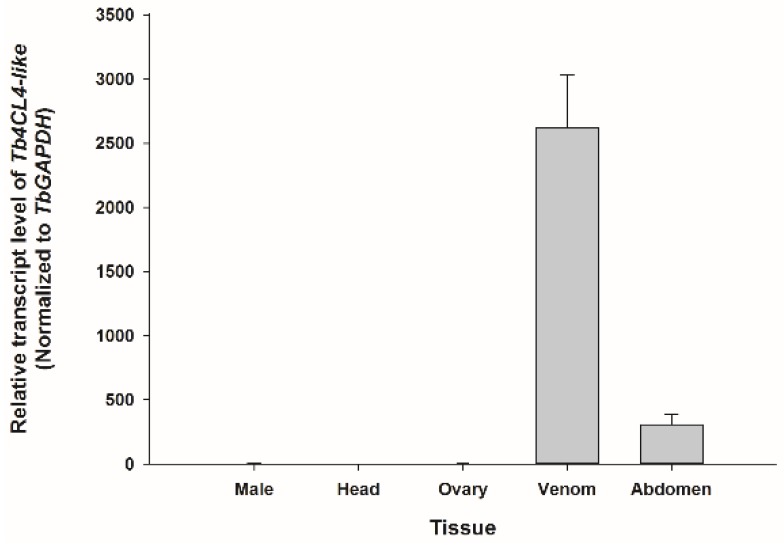
Expression profile analysis of Tb4CL4-like in *T. brontispae* female tissues and males. The transcript level was normalized to the glyceraldehyde-3-phosphate dehydrogenase (GAPDH) reference gene and the data from head tissue. Error bars indicate standard error of the mean from four independent biological replications.

To investigate the tissue distribution of *Tb4CL4-like* expression, qRT-PCR was performed using isolated tissues from females as well as whole body homogenates from male adults (Figure 3). The results showed that Tb4CL4-like was highly and specifically expressed in the venom apparatus. The expression levels in the remaining carcasses from female abdomens may be due to the broken venom apparatus during sample dissecting.

### 2.3. Tb4CL4-Like Expression, Purification and Western Blot Analysis

**Figure 4:**
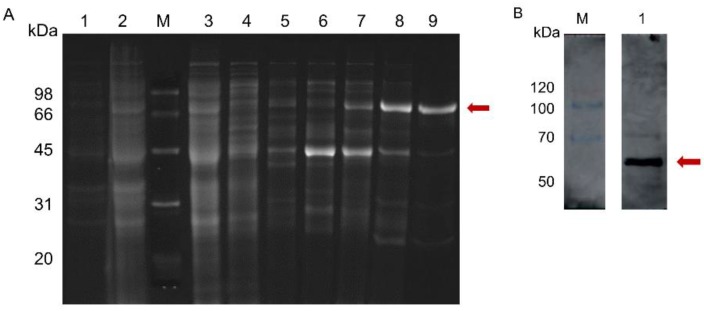
SDS-PAGE (**A**) and immunoblot (**B**) analyses of recombinant Tb4CL4-like. (A): Lane 1, uninduced protein; lane 2, induced protein; lane 3, flow-through of protein sample; lane 4, elution of equilibration buffer; lanes 5–9, elution buffer from 20, 40, 100, 120 and 140 mM imidazole buffer, respectively. (B): Lane 1, Tb4CL4-like-His fusion protein was analyzed by immunoblotting with anti-His as the primary antibody. The target band is marked in red. The positions and sizes (in kDa) of the weight standards in lane M are indicated.

The coding sequencing of mature Tb4CL4-like was cloned into the pET-28a vector and successfully expressed in BL21 (DE3). SDS-PAGE and Coomassie blue staining revealed a molecular weight estimate of 67 kDa, which corresponded to the Tb4CL4-like-His fusion protein partitioned into both the soluble and insoluble fraction (Figure S3). The soluble protein was then collected and purified under native conditions by Ni-nitrilotriacetic acid affinity chromatography. As shown in Figure 4, only a single predicted band was detected when purified Tb4CL4-like was analyzed by SDS-PAGE followed by Western blot using an anti-His tag antibody as the primary antibody. The recombinant Tb4CL4-like-His fusion protein was approximately 40 ng/μL.

### 2.4. Effect of Tb4CL4-Like on Host Cell Encapsulation Response

**Figure 5:**
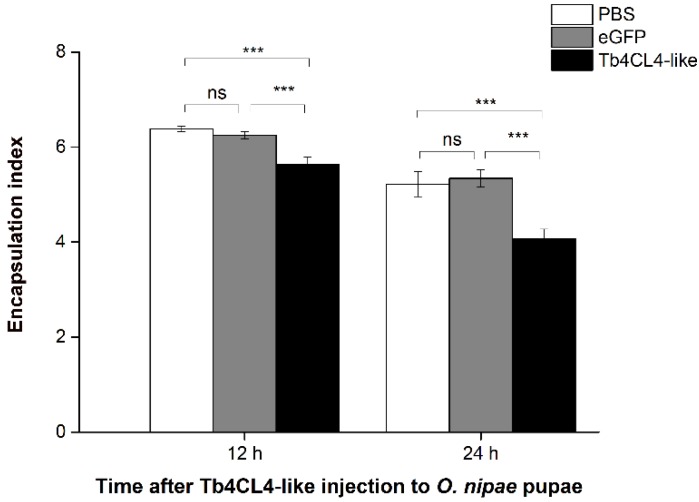
Host hemocyte encapsulation analysis by encapsulation index after injection of recombinant Tb4CL4-like. Hemolymph was collected from *Octodonta nipae* pupa at 12 or 24 h after injection of Tb4CL4-like. Pupae injected with phosphate buffered saline (PBS) or enhanced green fluorescent protein (eGFP) were used as a control. The encapsulation of beads was observed and recorded under a microscope. Data are represented as the means ± standard error, n = 5; *** and ns denote the significant difference of *p* = 0.001 and no significant difference, respectively.

To investigate the effect of the recombinant Tb4CL4-like-His fusion protein on the cell encapsulation response by host pupal hemocytes, in vitro encapsulation assays were performed using Sephadex DEAE A-50 beads. At 12 and 24 h after injection of enhanced green fluorescent protein (eGFP) or phosphate-buffered saline (PBS), beads tended to be strongly encapsulated by host pupal hemocytes with multiple layers. In contrast, the presence of the Tb4CL4-like-His fusion protein suppressed or reduced the encapsulation of beads. The encapsulation index and encapsulation rate clearly indicated that the Tb4CL4-like-His fusion protein had a significant effect on the host cell encapsulation response (12 h, *F*_2,12_ = 13.59, *p* = 0.001; 24 h, *F*_2,21_ = 9.378, *p* = 0.001). As shown in Figure 5, the encapsulation index in the treatment with the Tb4CL4-like-His fusion protein (5.6 and 4.1 at 12 and 24 h, respectively) was markedly lower than that of the treatment with eGFP (6.2 and 5.3 at 12 and 24 h, respectively) or PBS (6.4 and 5.2 at 12 and 24 h, respectively).

### 2.5. Effect of Tb4CL4-Like on Host Hemocyte Cytoskeleton, F-Actin Content and Spreading Ability

**Figure 6:**
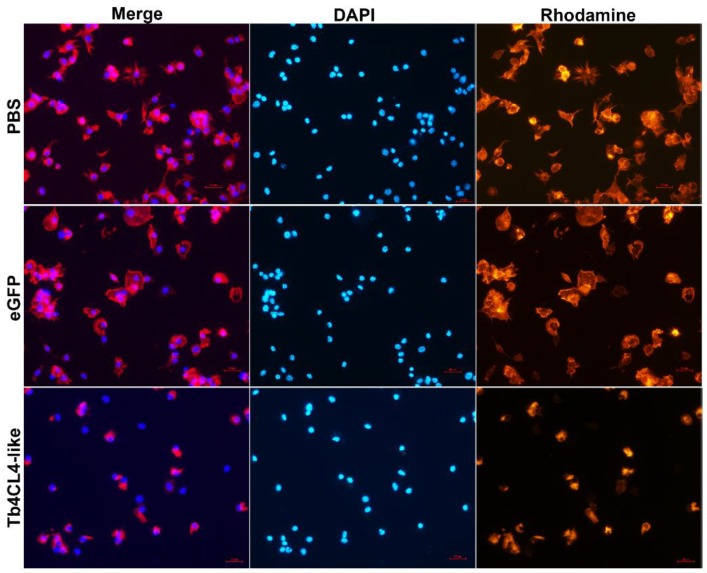
Comparison of hemocytes from Tb4CL4-like treated pupae to hemocytes from the control groups. Hemocytes were collected from *O. nipae* pupa at 24 h post injection of Tb4CL4-like. Pupae injected with PBS or eGFP were used as a control. Merge, merged images of the presented fluorescent channels; DAPI, hemocyte nuclei staining (blue); Rhodamine, hemocyte cytoskeleton stained with rhodamine–phalloidin (red). The bar shows a 20 µm scale.

**Figure 7:**
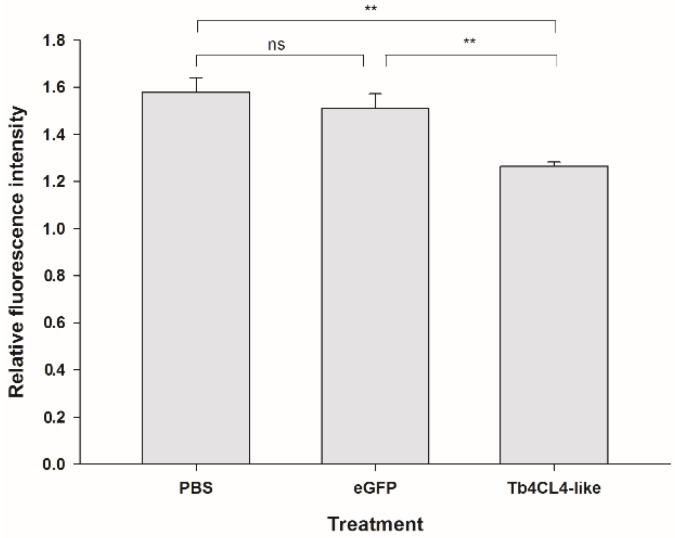
Effect of recombinant Tb4CL4-like on the F-actin content in host pupal hemocytes. Hemocytes were collected from *O. nipae* pupa at 24 h post injection of Tb4CL4-like and extracted by methanol. Data are represented as the means ± SE from four independent assays; ** and ns denote the significant difference of *p* < 0.01 and no significant difference, respectively.

It seemed that Tb4CL4-like had a more inhibitory effect on cellular immunity at 24 h (22.6%) after injection compared with that at 12 h (9.7%) based on the encapsulation; thus, we investigated the effect of Tb4CL4-like on the hemocyte cytoskeleton and F-actin content at 24 h post injection. Staining of hemocytes from the treatment with PBS or eGFP showed distinct differences in actin polymerization compared to treatment with the Tb4CL4-like-His fusion protein (Figure 6). Most of the hemocytes in the control sample were highly organized plasmatocytes and granulocytes. The plasmatocytes spread and extended pseudopods with a characteristic fibroblast-like morphology. The granulocytes were refractive and spread with shorter filopodia. Plasmatocytes were in an interdigitate junction with the adjacent cells by filopodia. In contrast, following the incubation of hemocytes with Tb4CL4-like in vivo for 24 h, the majority of hemocytes were in a rounded configuration, and some of them appeared to extend fewer and shorter pseudopods. The reduced spreading ability meant it was more difficult to distinguish plasmatocytes from granulocytes. In addition, it seemed that the number of the cells with only visible nuclei but without cytoplasm was increased with the Tb4CL4-like compared with that of the control group. Oenocytoids are usually rather fragile and quickly lyse in vitro; thus, these cells with only nuclei were likely to be oenocytoids. When quantifying the effect of Tb4CL4-like on the actin cytoskeleton, treatment with Tb4CL4-like led to a significant reduction in F-actin content in hemocytes compared with the injection of PBS or eGFP (*F*_2,6_ = 16.67, *p* < 0.01; Figure 7).

**Figure 8:**
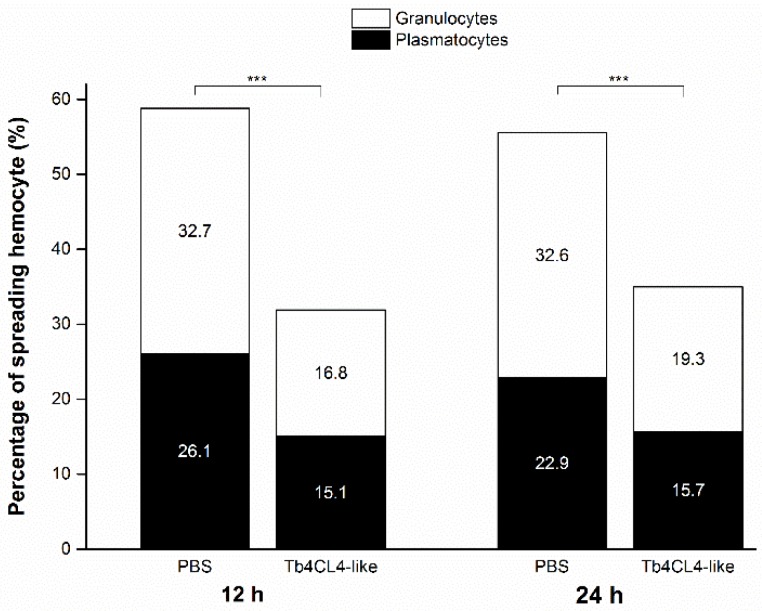
Inhibition of host pupal hemocyte spreading by recombinant Tb4CL4-like. Hemolymph was collected from *O. nipae* pupa at 12 or 24 h post injection of Tb4CL4-like and incubated onto a glass slide for 1 h. Pupae injected with PBS were used as a control. The attached hemocytes were observed and counted under a differential interference contrast microscope for spreading rate. A total of approximately 3000 hemocytes were analyzed for Tb4CL4-like or PBS. The values within each bar represent the proportion of spreading hemocytes that were plasmatocytes or granulocytes. *** denotes the significant difference of *p* < 0.001.

While nearly 60% of the plasmatocytes plus granulocytes from the treatment with PBS attached to the slide with lamellipodia or filopodia, only approximately 30% of the hemocytes spread after injection with the Tb4CL4-like-His fusion protein from 12 to 24 h (Figure 8; 12 h, *df* = 1, *χ*^2^ = 530.46, *p* < 0.001; 24 h, *df* = 1, *χ*^2^ = 407.10, *p* < 0.001). This reduction in spreading ability after injection with the Tb4CL4-like-His fusion protein could be observed well when hemocytes were stained with rhodamine–phalloidin (Figure 6).

### 2.6. Effect of Tb4CL4-Like on Phagocytosis

**Figure 9:**
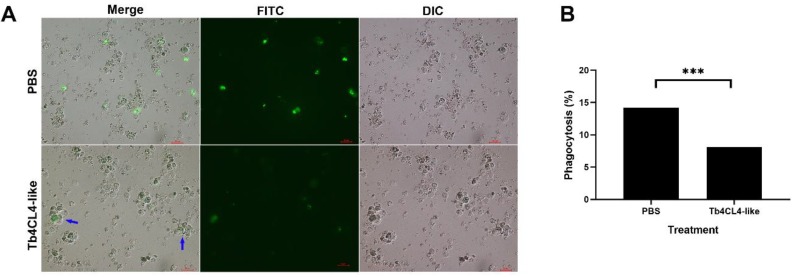
Qualitative (**A**) and quantitative (**B**) analyses of inhibition of host hemocyte phagocytosis by recombinant Tb4CL4-like. Hemocytes were collected from *O. nipae* pupae treated with Tb4CL4-like for 12 h followed by fluorescein isothiocyanate (FITC)-labeled *Escherichia coli* injection for another 12 h. Pupae injected with PBS were used as a control. (**A**): Aggregates containing blebbing hemocytes are marked with blue arrows. The bar shows a 20 µm scale. DIC, hemocytes observed under differential interference contrast microscope; FITC, *E. coli* labeled with FITC (green); Merge, merged images of FITC and DIC. (**B**): A total of approximately 2000 and 1600 hemocytes were analyzed for Tb4CL4-like and PBS, respectively. *** denotes the significant difference of *p* < 0.001.

When hemocytes were treated with Tb4CL4-like (40 ng/μL) and then incubated with *Escherichia coli*, the bacteria engulfed by granulocytes and plasmatocytes were significantly reduced (Figure 9A,B; *df* = 1, *χ*^2^ = 33.88, *p* < 0.001), and the percentage of phagocytosis was only half that of the treatment with PBS (Figure 9B). Although the phagocytosis of *E. coli* by plasmatocytes was observed, most of the injected *E. coli* was engulfed by granulocytes regardless of the treatment (Figure 9A). In addition, granulocytes from the treatment with Tb4CL4-like tended to cluster together into aggregates that contained blebbing hemocytes (Figure 9A, blue arrow).

### 2.7. Potential Binding Characteristics of Tb4CL4-Like

**Figure 10:**
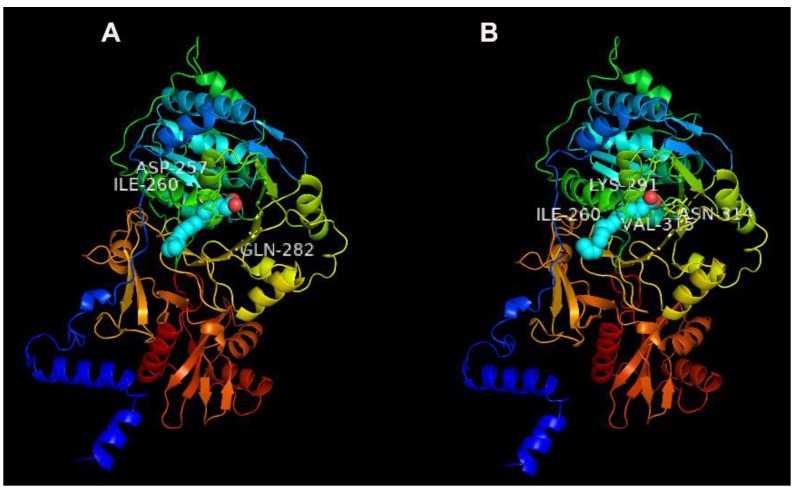
The complex and detailed binding mode of Tb4CL4-like with linolenic acid (**A**) and palmitic acid (**B**). The optimal ligand–protein complexes were chosen by their binding energy and inhibition constant (*K_i_*). Residues playing important roles in the binding are indicated.

Previous study revealed that luciferin 4-monooxygenase can catalyze the fatty acyl-CoA synthetic reaction. To understand whether Tb4CL4-like has this characteristic and is involved in fatty acid metabolism, the potential binding characteristics of Tb4CL4-like with seven long chain fatty acids were predicted using the molecular docking assay. The results showed that Tb4CL4-like exhibited greater affinity to palmitic acid and linolenic acid among the seven long chain fatty acids based on the values of binding energy and the inhibition constant *K*_i_ (Table 1). The molecular interactions between Tb4CL4-like and the two fatty acids are shown in Figure 10.Several residues playing important roles in the binding are indicated: Asp257 (H-bond), Ile260 and Gln282 (H-bond) for the binding of Tb4CL4-like and linolenic acid; Ile260, Lys291, Asn314 (H-bond) and Val315 for the binding of Tb4CL4-like and palmitic acid.

## 3. Discussion

Components including venom and ovarian/calyx fluid injected into the host at the time of depositing wasp eggs play key roles in facilitating successful parasitization and in creating a favorable environment for parasitoid development by altering host immunity and/or manipulating host development and physiology [1,4]. The abundant venom proteins appear to be most likely involved in parasitization success and thus attract the attention of investigators interested in the interaction between host and parasitoid. The alteration in hemocyte spreading, morphology and differential hemocyte counts are essential parts of the host cellular response against endoparasitoids. Here, we investigated the physiological effects of a new venom protein, Tb4CL4-like, on host hemocyte behavior and the possible mechanism of action in host immunosuppression.

4CL is widespread in plants; thus, we previously regarded Tb4CL4-like identified in the venom reservoir of *T. brontispae* as a result of lateral gene transfer [20]. However, based on the whole genome shotgun sequence, 4CL has also been identified in insects, such as Hymenoptera, Coleoptera and Lepidoptera (Figure S4). Although we nominated this venom protein as 4CL4-like, the phylogenetic tree in Figure 2 revealed that it shows greater similarity to an uncharacterized protein. In addition, luciferin 4-monooxygenase, also named firefly luciferase, is also a member of the Class I adenylate-forming enzymes and possesses a bifunction of catalyzing not only the bioluminescence reaction but also the fatty acyl-CoA synthetic reaction [25]. We performed a molecular docking of Tb4CL4-like with various long chain fatty acids and found that Tb4CL4-like exhibited greater affinity to palmitic acid and linolenic acid. On the basis of these analyses, the name “luciferin 4-monooxygenase” may be better than that of “4CL4” for Tb4CL4-like, and Tb4CL4-like may serve to synthesize a long chain acyl-CoA in the activation step that ultimately leads to degradation of fatty acids.

Venom proteins may be specifically or abundantly transcribed in venom tissues. For example, the transcript levels of two Kazal-type serine protease inhibitors and a small pacifastin protease inhibitor from *N. vitripennis* were mostly detected in the venom apparatus [26,27]. The mRNA level of venom calreticulin from *Pteromalus puparum* was also significantly higher in the venom gland than in other tissues, although this venom protein was partly secreted as a component in the venom [13]. Similarly, in the present study, Tb4CL4-like was transcribed mainly in the venom apparatus of *T. brontispae*, in which the transcripts were more than five hundred times higher than those in other tissues tested (excluding the abdomen carcass because of the leakage of venom during sample collection). Moreover, the predicted secretory signal peptide was detected at the *N*-terminus of the Tb4CL4-like amino acid sequence. We thus inferred that Tb4CL4-like mRNA is transcribed in the venom gland, and its protein is partially or entirely secreted in the venom and deposited in the venom reservoir before its injection into host hemocoel.

With regard to evaluating the role of Tb4CL4-like in the modulation of host physiology, the recombinant Tb4CL4-like was expressed and purified. It was observed that the recombinant Tb4CL4-like suppressed the in vitro encapsulation of Sephadex DEAE A-50 beads by host hemocytes and their normal spreading behavior. As previously indicated, encapsulation of foreign targets relies on hemocyte cooperation and cytoskeleton rearrangement, which enable hemocytes to extend pseudopods and move [2,10,28,29]. In view of this, the effects of Tb4CL4-like on the hemocyte cytoskeleton were also investigated by visualizing as well as measuring F-actin. The results revealed that the recombinant Tb4CL4-like protein induced a disruption in the cytoskeleton of *O. nipae* hemocytes, which led to hemocytes incapable of sufficient organization and ultimately the reduction in forming pseudopods. We previously found that parasitization by *T. brontispae* and its venom under the concentration of one equivalent did not affect the total hemocyte counts and differential hemocyte counts of the host [19,30]. Therefore, Tb4CL4-like may hinder the encapsulation response as well as the spreading behavior by targeting the host hemocyte cytoskeleton. Until now, limited documents have reported that virulent factors, including polydnavirus, ovarian proteins and venom proteins from parasitoids, are responsible for depolymerization of actin in hemocytes. For instance, polydnavirus CrV1 from *Cotesia rubecula* and the CrV1-homolog from *Cotesia congregata* disrupted the cytoskeleton, spreading and adhesion of host hemocytes [31,32]. Ovarian protein of *Cotesia plutellae* significantly inhibited hemocyte spreading and F-actin development in the hemocytes of *Plutella xylostella* [33]. The venom protein VPr1 from *P. hypochondriaca* disrupted the organization of the host hemocyte cytoskeleton [10]. In contrast, although a 24 kDa venom protein from *P. puparum* could suppress the spreading behavior and encapsulation ability of host hemocytes [34], the venom of this parasitoid did not destroy the host hemocyte cytoskeleton [35].

Phagocytosis is a widely conserved defense in insects. Phagocytosis engulfs the target via actin polymerization-dependent mechanisms followed by maturation of the phagosome into a phagolysosome [2]. A previous report showed that preincubation of host hemocytes with the venom protein VPr1 from *P. hypochondriaca* reduced the percentage of phagocytosis, possibly due to the reduction in the host hemocyte number per millilitre caused by VPr1 [10]. The phagocytic assays performed in the present work also demonstrated that the treatment of host hemocytes with recombinant Tb4CL4-like resulted in a decrease in hemocyte phagocytic activity compared with the PBS treatment. Moreover, granulocytes that tended to cluster together into aggregates contained blebbing hemocytes. It is thus likely that this reduction in the percentage of phagocytosis may be attributed to the disruption of the host hemocyte cytoskeleton or the disintegration of granulocytes.

Tb4CL4-like is hypothesized to be involved in fatty acid metabolism. However, the immunosuppression effects of Tb4CL4-like on host cellular immunity, including hemocyte encapsulation, F-actin, spreading and phagocytosis, were confirmed in this study. This phenomenon can be interpreted as follows: it has been recently shown that fatty acid metabolism-derived signaling plays a crucial role in mediating insect immunity. Upon microbial pathogen infection, non-self recognition signals are propagated to hemocytes and/or fat bodies, in which eicosanoid signals can act as the ultimate downstream mediator [36]. For example, prostaglandins mediate actin filament-bundling in conjunction with F-actin formation to drive hemocyte-spreading behavior, which is involved in phagocytosis, nodulation, and encapsulation [37,38]. Therefore, it is not surprising to observe the immunosuppression effects of Tb4CL4-like on host cellular immunity. The future challenges will be to reveal the long chain fatty acyl-CoA synthetase activity of Tb4CL4-like and to explore its physiological effects on host fatty acid metabolism.

## 4. Materials and Methods

### 4.1. Insect Rearing

Laboratory cultures of the endoparasitoid *T. brontispae* and its host *O. nipae* were maintained at 25 ± 1 °C, 80% ± 5% relative humidity, and a photoperiod of 12:12 h (light:dark) as previously described [18,39]. After emergence, the adult wasps were collected and placed in plastic containers with hosts, and the wasps were fed a 10% (*v/v*) sucrose solution to prolong longevity. *O. nipae* was reared on the Canary Island date palm *Phoenix canariensis* Hort. ex young leaves. For the wasp propagation, one-day-old *O. nipae* pupae were provided for parasitization with about five times the number of wasps.

### 4.2. Cloning the Full-Length of the Tb4CL4-Like Gene

Total RNA was extracted from whole body homogenates of female wasps (without parasitization experience) using TRIzol reagent (Invitrogen, Carlsbad, CA, USA) according to the manufacturer’s protocol, and the concentration of RNA was determined by NanoDrop 2000 (Thermo Fisher Scientific, Billerica, MA, USA) and agarose gel electrophoresis. The RNA was then subjected to cDNA synthesis using the PrimeScript first cDNA Synthesis Kit (Takara, Dalian, China). The fragment homologous to 4CL4 was chosen from the *T. brontispae* transcriptome database [20] and validated by PCR using the 2× Taq Plus Master kit (Tiangen, Beijing, China). The obtained PCR products were purified and cloned into the pEASY-T1 simple cloning vector (TransGen, Beijing, China) before submission to Sangon Biotech Company (Shanghai, China) for sequencing. After sequence validation, the 5′ and 3′ cDNA ends of Tb4CL4-like were produced using the SMARTer^TM^ RACE cDNA Amplification kit (Takara) according to the manufacturer’s instructions. The rest of the cloning and sequencing of the RACE-PCR products were performed as described above. All primers were designed using Primer Premier 5.0 and are summarized in Table S3.

### 4.3. Sequence Analysis

The potential open reading frame, molecular weight and isoelectric point were analyzed with the DNAMAN 8.0.8.789 program. The signal peptide and conserved domains of the deduced amino acid sequence were detected with the SignalP 4.1 Server (https://www.cbs.dtu.dk/services/SignalP/) and the National Center for Biotechnology Information Conserved Domain Search (https://www.ncbi.nlm.nih.gov/Structure/cdd/wrpsb.cgi), respectively. The putative *N*-linked and *O*-linked glycosylation sites were predicted using the NetNGlyc 1.0 and NetOGlyc 4.0 Servers, respectively. The similarity of the Tb4CL4-like sequence to other proteins was performed with the BLAST tool (https://www.uniprot.org/blast/). Multiple alignments of 4CL amino acid sequences were conducted using ClustalW with default parameters, and their conserved motifs were further compared with the MEME online program (http://meme-suite.org/tools/meme). The phylogenic tree was constructed by the maximum likelihood method of MEGA X software with the following settings: Jones Taylor Thornton model and 1000 bootstrap replications. All amino acid sequences for alignment were acquired from UniProt. The names and accession numbers are summarized in Table S2.

### 4.4. Quantitative Real-Time PCR (qRT-PCR) Analysis of the Tb4CL4-Like Tissue Expression Profile

To investigate whether *Tb4CL4-like* was only transcribed in the venom apparatus, total RNA was extracted from the venom apparatus, the ovaries, the remaining carcasses from 500 female abdomens, the mix of head and thorax, as well as the whole body homogenates from 300 male adults using TRIzol reagent as described above. Tissues were collected with four biological replicates. Approximately 1 μg of total RNA was synthesized using the PrimeScript™ RT Reagent kit with gDNA Eraser (Takara). qRT-PCR was performed in triplicate for each biological replicate on the 7500 Real Time PCR System (Thermo Fisher Scientific) using PowerUp™ SYBR^®^ Green Master Mix (Thermo Fisher Scientific) with the same protocol as described previously [40]. The *T. brontispae* glyceraldehyde-3-phosphate dehydrogenase (TbGAPDH) gene was applied as an internal control to normalize the potential variation in cDNA samples. The standard curves for Tb4CL4-like and TbGAPDH were prepared by 5× serial dilutions of the cDNA samples. The expression levels were calculated with the accompanying ABI 7500 system software (V2.0.6). The primers for qRT-PCR are also provided in Table S3.

### 4.5. Expression and Purification of Recombinant Tb4CL4-Like

Specific primers containing *Sac*I and *Not*I restriction enzyme sites were designed to amplify the full fragment encoding the mature protein (Table S3). The PCR product was purified and inserted into the pEASY-T1 simple cloning vector. The recombinant pEASY-T1-Tb4CL4-like plasmid was digested with *Sac*I and *Not*I and ligated into pET-28a digested with *Sac*I/*Not*I, and the constructed plasmid was transformed into *Escherichia coli* BL21 (DE3) cells. Positive clones containing the inserted gene sequence were selected and confirmed by sequencing and then cultured in LB-kanamycin medium at 37 °C until the OD_600_ value ranged from 0.6–0.8. Recombinant protein expression was subsequently induced with the addition of isopropyl-β-D-thiogalactopyranoside (IPTG) to a final concentration of 0.8 mM and cultured at 16 °C for 18 h. After that, *E. coli* cells were harvested by centrifugation at 10,000× *g* at 4 °C for 2 min, rinsed with phosphate-buffered saline (PBS) (pH 7.0) three times, lysed with ultrasonication, and centrifuged. The resulting recombinant protein in the supernatant was collected and purified with a Ni-NTA affinity column (TransGen) according to the manufacturer’s instructions. The eluted fractions containing Tb4CL4-like were dialyzed against PBS overnight to obtain the purified protein.

### 4.6. SDS-PAGE and Immunoblot Analysis

Protein concentration was determined with the Bradford assay kit (TransGen) and then concentrated to a proper range using ultrafiltration (Amicon Ultra 10 kDa, Millipore, Darmstadt, Germany) facilitated by centrifugation. The purified proteins were separated on a 10% SDS-PAGE gel and then transferred to a nitrocellulose filter membrane using Trans-Blot SD (Bio-Rad, Hercules, CA, USA). The membrane was blocked for 1 h at 37 °C and then washed. Immunoblots were analyzed with anti-6×His antibody (diluted at 1:3000, Sangon Biotech) as the primary antibody and horseradish peroxidase-conjugated goat anti-rabbit IgG (diluted at 1:5000, Sangon Biotech) as the second antibody. The immunoblot signal was detected using Immobilon Western Chemiluminescent HRP Substrate (Millipore) on an Amersham Imager 600 QC (GE Healthcare, Boston, MA, USA).

### 4.7. In Vitro Encapsulation Assay

Encapsulation assays were performed with *O. nipae* hemocytes in vitro as described by Hu et al. [41]. Briefly, one-day-old *O. nipae* pupa was injected with 207 nL of PBS, eGFP (120 ng/μL, Solarbio, Bejing, China) or Tb4CL4-like (40 ng/μL) using a NANOLITER 2010 (World Precision Instruments, Sarasota, FL, USA). Approximately 12 and 24 h later, 4 μL of mixed hemolymph from 5–6 pupae from a same batch was mixed with 6 μL of Schneider medium (Thermo Fisher Scientific) in a 200-μL PCR tube containing 10 Sephadex DEAE A-50 beads (<150 μm in diameter) stained with 0.1% congo red. Tubes containing hemolymph were attached to a revolver (TR-02U, Crystal Industry, Dallas, TX, USA) rotating at a speed of 10 rpm to keep the beads in contact with hemocytes for 24 h at room temperature. Beads were then placed on slides to be observed and recorded under a differential interference contrast microscope (DIC, Nikon, Tokyo, Japan) and imaged with the accompanying NIS-Elements D 4.30.00 software. Five replicates were included in each treatment.

To compare the extent of the encapsulation, two parameters, including the encapsulation index and encapsulation response, were adopted. For the encapsulation index, first, beads were assigned to five (0–4) and seven (0–6) grades based on the area and the thickness of the capsule, respectively, as described previously with some modifications [42,43]. The area of the capsule (*A*) = total degree of angle covering the beads/360: 0, *A* = 0; 1, *A* = 0–0.25; 2, *A* = 0.25–0.5; 3, *A* = 0.5–0.75; 4, *A* = 0.75–1.0. The thickness of the capsule (*T*) = (SQRT (Area/π)-Radius _beads_)/Radius _beads_: 0, *T* = 0; 1, *T* = 0–0.2; 2, *T* = 0.2–0.4; 3, *T* = 0.4–0.6; 4, *T* = 0.6–0.8; 5, *T* = 0.8–1.0; 6, *T* = 1.0–2.0. The encapsulation index was calculated as follows = Σ (*P* × *T* + *P* × *A*), where *P* represents the percentage of beads with a defined grade.

### 4.8. Effect of Tb4CL4-Like on F-Actin of Hemocytes

F-actin structure was visualized according to the protocol previously described with modifications [33]. Hemolymph was collected 24 h post injection of PBS, eGFP or Tb4CL4-like as described in the above section. Approximately 3 μL of mixed hemolymph from 5–6 pupae from the same batch was mixed with 2 μL of Pringle’s saline and then placed on a glass slide coated with polylysine. Hemocytes were allowed to attach for 40 min in a humid chamber, washed, fixed with 4% paraformaldehyde in PBS for 15 min and washed in PBS again. The treated hemocytes were then incubated with 5 μL of 0.1% Triton X-100 for 5 min, washed and blocked with 20 μL of 1% BSA for 1 h at room temperature. Afterwards, an aliquot of 10 μL of 0.33 μM rhodamine–phalloidin (Invitrogen) was applied, and the hemocytes were incubated for 30 min in the dark at room temperature. The glass slides were washed thoroughly with PBS, and 10 μL of 1 μg/μL 4′,6-diamidino-2-phenylindole 4′,6-diamidino-2-pheny-lindole (DAPI) was added to the hemocytes and incubated for another 10 min in the dark. The hemocytes were washed with PBS again and then visualized under a fluorescence microscope (Nikon).

To investigate the effect of Tb4CL4-like on F-actin more accurately, the F-actin content was quantified based on the phalloidin binding technique as described previously with some modifications [33]. An aliquot of 4 μL of mixed hemolymph from 5-6 pupae from a same batch was diluted with 20 μL of Pringle’s saline and then centrifuged at 1500 g at 4 °C for 10 min. The washed hemocyte suspension was fixed with the addition of 20 μL of 4% paraformaldehyde in PBS for 15 min and washed three times by centrifugation using PBS. After that, 40 μL of 0.55 μM rhodamine–phalloidin was added to the hemocytes and incubated for 30 min in the dark at 4 °C, and the hemocytes were washed by centrifugation again. The bound phalloidin was subsequently solubilized with 300 μL of methanol by frequent vortexing for 1 h in the dark. The hemocytes were centrifuged at 14,000 g for 5 min, and their fluorescence intensity was measured in an F-4600 spectrofluorometer (Hitachi, Tokyo, Japan) (excitation at 540 nm, emission at 565 nm). For blank samples, 4 μL of Pringle’s saline in place of hemolymph was used. Relative fluorescence intensity was calculated by dividing the fluorescence intensity of the treated sample by that of the blank sample. Four biological replicates were performed for each treatment.

### 4.9. Hemocyte Spreading Assay

The treatment with eGFP showed similar effect on host encapsulation and F-actin content compared with PBS based on the above results, therefore in the following experiment, we used treatment of PBS only as a control. Hemolymph was collected 12 or 24 h post injection of PBS or Tb4CL4-like as described in the above section. Approximately 4 μL of hemolymph was mixed with 6 μL of Pringle’s saline and then pipetted onto a glass slide in a humid chamber. After incubation for 1 h, the attached hemocytes were washed in PBS, fixed with 4% paraformaldehyde in PBS for 30 min and washed in PBS again. Spreading and nonspreading hemocytes were observed under the differential interference contrast microscope with the criteria specified by Meng et al. [30]. Each treatment (containing 500 hemocytes in total) was repeated six times. The percentage of spreading hemocytes was calculated as the number of spreading plasmatocytes plus granulocytes observed divided by the total number of plasmatocytes and granulocytes observed and multiplied by 100%.

### 4.10. In Vivo Phagocytosis Assay

Fluorescein isothiocyanate (FITC)-labeled *E. coli* at a concentration of 2.0 × 10^8^ cells/mL was generated as previously described by Meng et al. [30]. In the phagocytosis assay, a one-day-old *O. nipae* pupa was injected with 207 nL of PBS or Tb4CL4-like (40 ng/μL). Following incubation for 12 h, 138 nL of heat-killed FITC-labeled bacteria was injected into the same pupa and incubated for another 12 h. Afterwards, an aliquot of 3 μL of hemolymph from five pupae was added to a tube containing 2 μL of Pringle’s saline, and the mixture was placed on a glass slide coated with polylysine. Hemocytes were allowed to attach for 30 min in a humid chamber, washed with PBS, and observed under a fluorescence microscope. The number of hemocytes ingesting one or more *E. coli* was recorded, and a total of 400–600 hemocytes were examined for each treatment. Assays were repeated in triplicate.

### 4.11. Molecular Docking of Tb4CL4-Like

Putative binding properties of Tb4CL4-like with seven long chain fatty acids (Table 1) was predicted using a molecular docking assay with AutoDock 4.2.6 which applies the Lamarckian genetic algorithm (LGA) as previously described [44]. A rigid macromolecule-flexible ligand docking method was used. The protein 3D-structure prediction of Tb4CL4-like was acquired by the I-TASSER server (http://zhanglab.ccmb.med.umich.edu/I-TASSER/, Model 4 with C-score = 0.14). The 3D-structure of the seven long chain fatty acids was obtained from the PubChem chemical database (https://pubchem.ncbi.nlm.nih.gov). The grid box was generated using the AutoGrid program using the following settings: *x*–, *y*– and *z*–axes all of 126 Å grid points, and grid spacing of 0.375 Å. LGA was used for the docking calculations. AutoDock was run with default parameters. Docked conformation with lowest binding energy out of ten different conformers was selected for further analysis. Docking results were decorated by PyMOL 2.3.0 to show the 3D protein–ligand complex. The ligand–protein docked complexes were selected based on the binding energy and inhibition constant (*K*_i_).

### 4.12. Statistical Analysis

Data were expressed as the mean ± standard error and analyzed using SPSS 20.0 for windows. Differences among treatments for the values of encapsulation index and F-actin content (relative fluorescence intensity) were examined by one-way ANOVA and Tukey’s test because of the homogeneity of variance at statistical significance of *p* < 0.05. The spreading ratio and phagocytosis ratio between treatments were analyzed by chi-square test at statistical significance of *p* < 0.001.

## Figures and Tables

**Table 1:** Putative binding mode of Tb4CL4-like with seven long chain fatty acids.

Ligands	Tb4CL4-Like	
	Binding Energy (kcal/mol)	*K_i_* (μM)
Arachidonic acid	−8.06	1.23
Caffeic acid	−6.40	20.27
Ferulic acid	−6.47	18.03
Linoleic acid	−6.37	21.39
Linolenic acid	−8.35	0.72
Palmitic acid	−8.69	0.43
*p*-Coumarate	−6.41	19.92

*K_i_*: Inhibition constant.

## Supplementary Materials

The following are available online at https://www.mdpi.com/2072-6651/11/11/672/s1: Figure S1: Nucleotide and amino acid sequence of *T. brontispae* 4CL4-like. The predicted secretion signal peptide is shaded. A polyadenylation signal near the 3′ end is underlined. The substrate binding sites and catalytic sites are marked by “■” and “▲”, respectively. Putative *N*-linked and *O*-linked glycosylation sites are labeled with “□” and “○”, respectively; Figure S2: A section of sequence alignments of Tb4CL4-like and 21 proteins that show more than 25% identity to Tb4CL4-like from the Universal Protein Resource (UniProt). Accession numbers of the sequences are listed in Table S2; Figure S3: Expression of recombinant Tb4CL4-like induced by different concentrations of isopropyl-β-D-thiogalactopyranoside (IPTG) at 16 °C (A) and analysis of its solubility (B). (A): Lane 1, no IPTG induction; lanes 2–4, concentration of IPTG was 0.5, 0.8 and 1 mM, respectively. (B): Lane 1, no IPTG induction; lane 2, homogenate after IPTG induction; lane 3, supernatant after IPTG induction; lane 4, precipitate after IPTG induction. The positions and sizes (in kDa) of the weight standards in lane M are indicated. The target band is marked by a red arrow; Figure S4: Phylogenetic relationships of Tb4CL4-like and other Class I adenylate-forming enzyme members from plants and insects. The amino acid sequences of the complete proteins were aligned with the BLAST tool (https://blast.ncbi.nlm.nih.gov/Blast.cgi) to construct the fast minimum evolution tree (Max seq difference, 0.85; Distance, Grishin protein). Tb4CL4-like and plants are shaded in yellow and purple, respectively; Table S1: Analysis and distribution of conserved motifs in the 21 proteins; Table S2: Information of 21 proteins selected from UniProt; Table S3: Primers used in the study.

## Data and Materials Availability

DNA sequence of Tb4CL4-like was deposited in Genbank with accession number MN518767. Correspondence for materials should be addressed to B.-Z.T.
