# Supplementary material for: Cloning and Immunosuppressive Properties of an Acyl-Activating Enzyme from the Venom Apparatus of Tetrastichus brontispae (Hymenoptera: Eulophidae)
Source: Toxins (Basel). 2019 Nov 18;11(11):672. doi: 10.3390/toxins11110672 (PMC6891662; doi:10.3390/toxins11110672)
Supplement: Supplementary file 1 [file toxins-11-00672-s001.zip › toxins-617646-SI.docx]

Supplementary Materials: Cloning and Immunosuppressive Properties of an Acyl-Activating Enzyme from the Venom Apparatus of *Tetrastichus brontispae* (Hymenoptera: Eulophidae)

Xiao-Mei Zhang, Hua-Jian Zhang, Min Liu, Bin Liu, Xia-Fang Zhang, Cheng-Jun Ma, Ting-Ting Fu, You-Ming Hou and Bao-Zhen Tang


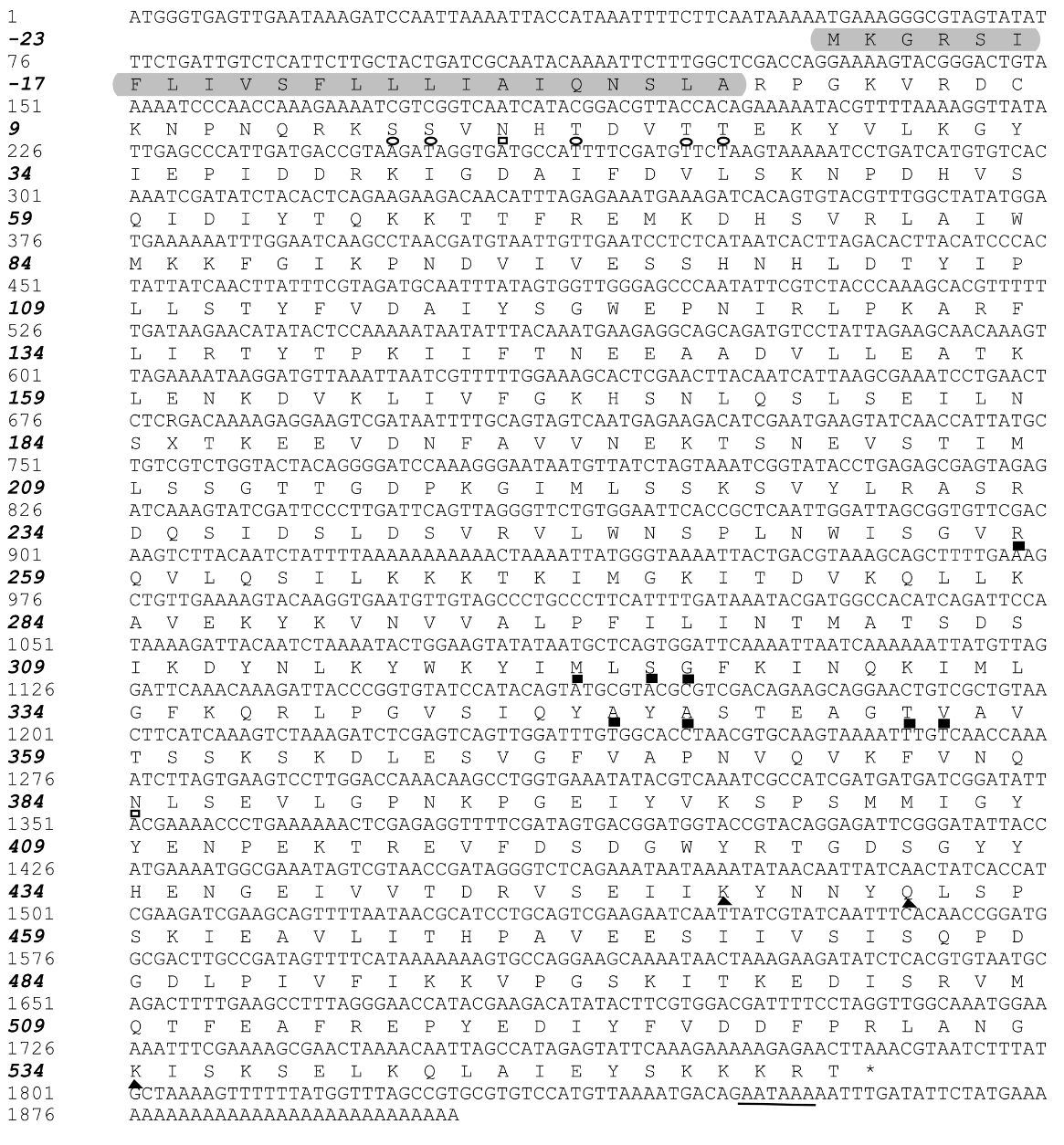


**Figure S1.** Nucleotide and amino acid sequence of *T. brontispae* 4CL4-like. The predicted secretion signal peptide is shaded. A polyadenylation signal near the 3′ end is underlined. The substrate binding sites and catalytic sites are marked by “■” and “▲”, respectively. Putative *N*-linked and *O*-linked glycosylation sites are labeled with “□” and “○”, respectively.


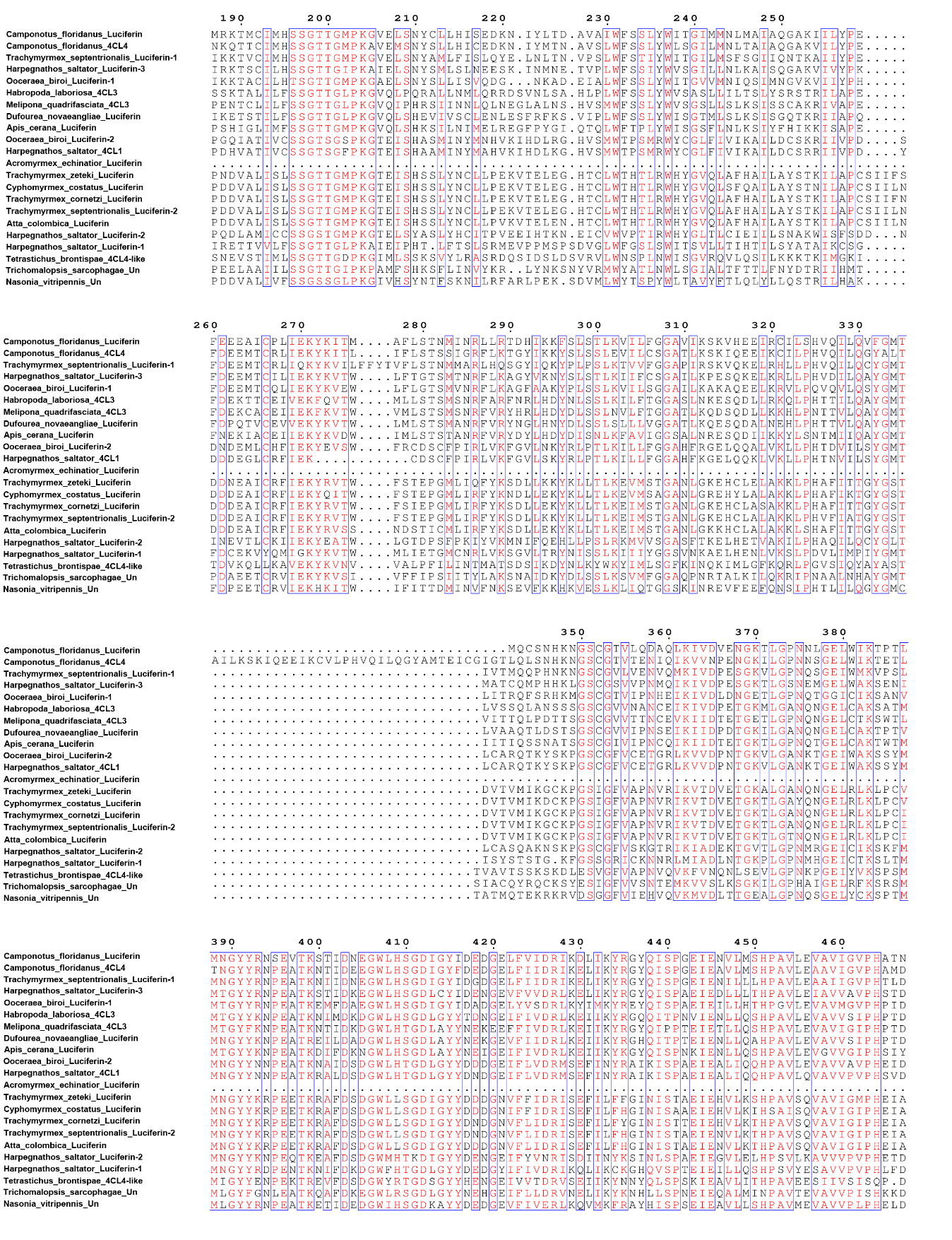


**Figure S2.** A section of sequence alignments of Tb4CL4-like and 21 proteins that show more than 25% identity to Tb4CL4-like from the Universal Protein Resource (UniProt). Accession numbers of the sequences are listed in Table S2.


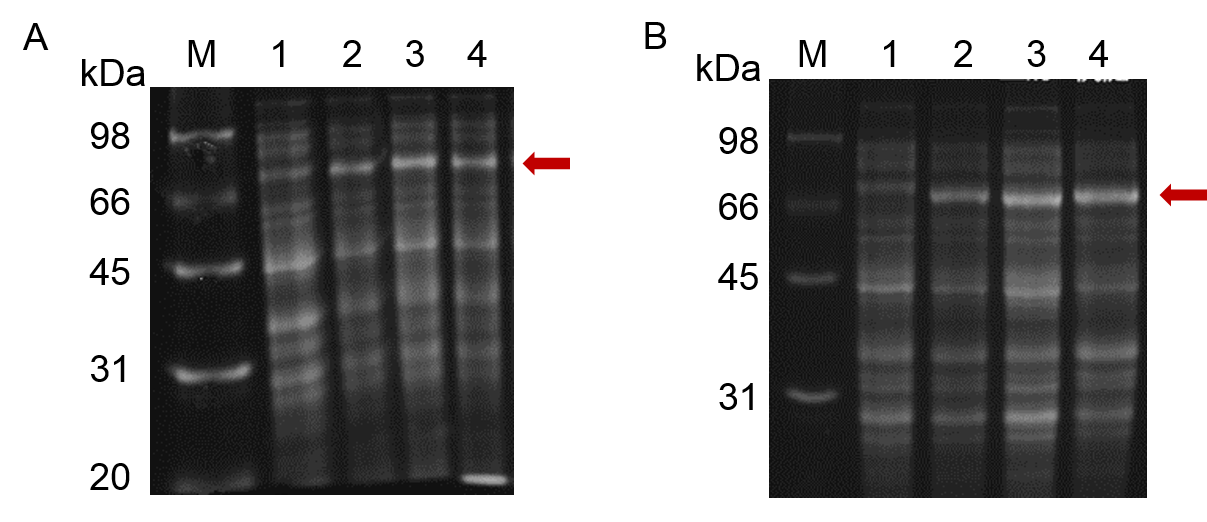


Figure S3: Expression of recombinant Tb4CL4-like induced by different concentrations of isopropyl-β-D-thiogalactopyranoside (IPTG) at 16 °C (**A**) and analysis of its solubility (**B**). (**A**): Lane 1, no IPTG induction; lanes 2–4, concentration of IPTG was 0.5, 0.8 and 1 mM, respectively. (**B**): Lane 1, no IPTG induction; lane 2, homogenate after IPTG induction; lane 3, supernatant after IPTG induction; lane 4, precipitate after IPTG induction. The positions and sizes (in kDa) of the weight standards in lane M are indicated. The target band is marked by a red arrow.


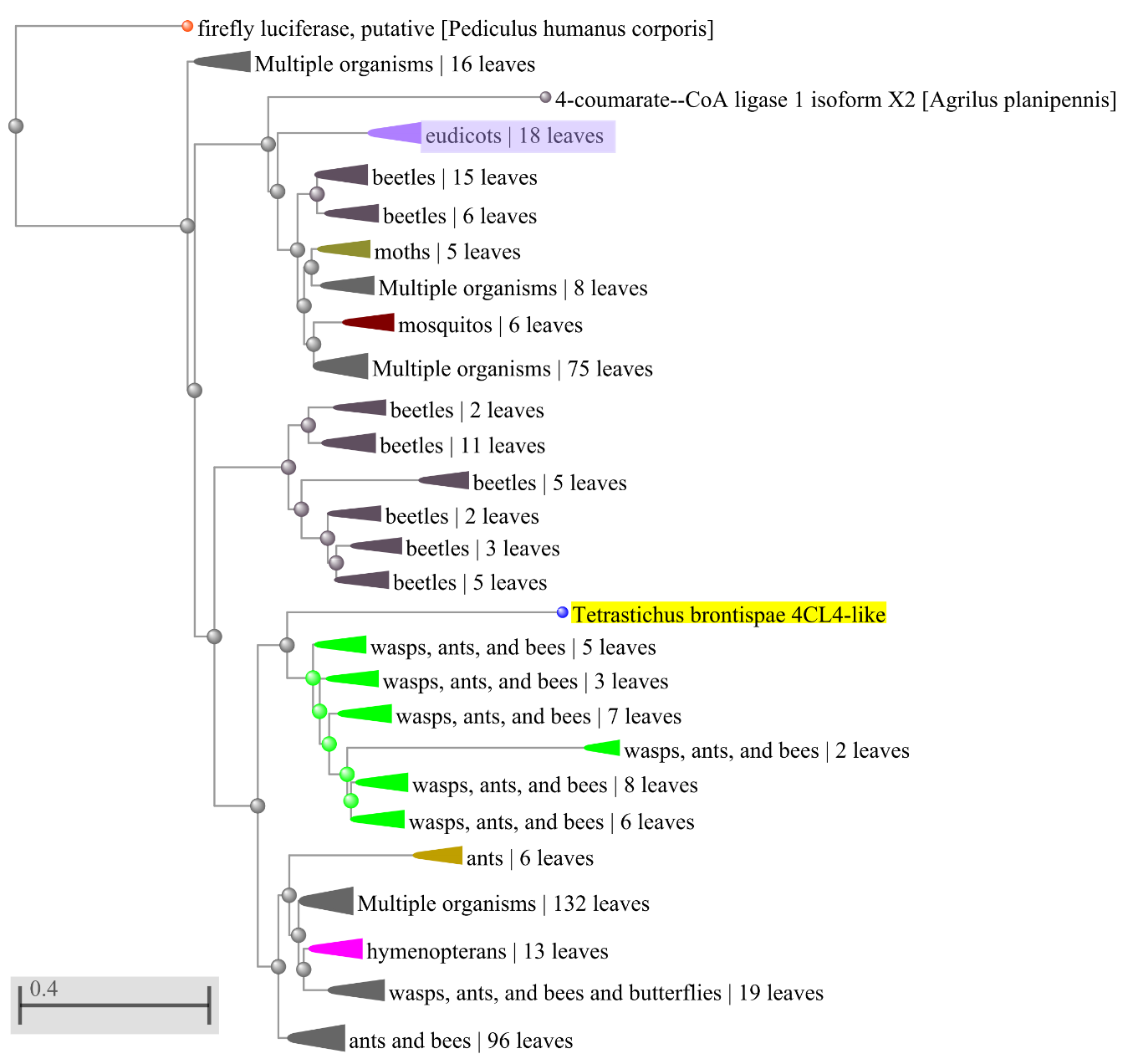


**Figure S4.** Phylogenetic relationships of Tb4CL4-like and other Class I adenylate-forming enzyme members from plants and insects. The amino acid sequences of the complete proteins were aligned with the BLAST tool (https://blast.ncbi.nlm.nih.gov/Blast.cgi) to construct the fast minimum evolution tree (Max seq difference, 0.85; Distance, Grishin protein). Tb4CL4-like and plants are shaded in yellow and purple, respectively.
